# Digital health literacy in Norwegian patients with hip and knee arthroplasty: normative data from a cross-sectional study

**DOI:** 10.2340/17453674.2024.42304

**Published:** 2024-11-25

**Authors:** Turid ROGNSVÅG, Ingrid K NORDMO, Ingvild B BERGVAD, Anne M FENSTAD, Ove FURNES, Anners LERDAL, Maren F LINDBERG, Søren T SKOU, Mona BADAWY

**Affiliations:** 1 Coastal Hospital in Hagevik, Department of Orthopedic Surgery, Haukeland University Hospital, Bergen, Norway; 2 Department of Clinical Medicine, University of Bergen, Bergen, Norway; 3 Surgical Department, Lovisenberg Diaconal Hospital, Oslo, Norway; 4 Department of Interdisciplinary Health Sciences, Institute of Health and Society, Faculty of Medicine, University of Oslo, Oslo, Norway; 5 Research Department, Lovisenberg Diaconal Hospital, Oslo, Norway; 6 The Norwegian Arthroplasty Register, Department of Orthopedic Surgery, Haukeland University Hospital, Bergen, Norway; 7 Department of Public Health Science, Institute of Health Science, Faculty of Medicine, University of Oslo, Oslo, Norway; 8 Center for Muscle and Joint Health, Department of Sports Science and Clinical Biomechanics, University of Southern Denmark, Odense, Denmark; 9 The Research and Implementation Unit PROgrez, Department of Physiotherapy and Occupational Therapy, Næstved–Slagelse–Ringsted Hospitals, Slagelse, Denmark

## Abstract

**Background and purpose:**

As digital health services become increasingly important in osteoarthritis treatment, understanding patients’ digital health literacy (eHL) is crucial, including those undergoing total hip and knee arthroplasty (THA/TKA). We primarily aimed to provide eHL norms in a representative group of Norwegian patients, and secondarily to examine the relationships between eHL and health-related quality of life (QoL).

**Methods:**

We invited 800 randomly selected THA/TKA patients from the Norwegian Arthroplasty Register to complete a paper-based questionnaire, which included sociodemographic variables. eHL was measured using the eHealth Literacy Questionnaire (eHLQ) with 7 domains: Using technology, Understand, Engage, Control, Motivation, Access, and Needs, scored from 1 (strongly disagree) to 4 (strongly agree). The EuroQol EQ-5D-5L measured health-related QoL. We used multivariable regression to examine relationships between eHL domains and health-related QoL controlling for sociodemographic variables.

**Results:**

Respondents’ (N = 383, 48%) mean age was 70 years (SD 9.0) and 246 (64%) were female. Mean eHLQ and the proportion of patients with low eHL (≤ 2.5) were Technology 2.7 (34%), Understanding 3.0 (14%), Engage 2.7 (28%), Control 3.2 (7.7%), Motivation 2.8 (35%), Access 2.8 (33%), and Needs 2.6 (46%). Low eHL correlated with older age and low education, but not with sex or type of surgery. Regression analyses showed that lower scores on the domains Technology, Engage, Control, Access, and Needs were associated with poorer QoL after adjusting for sociodemographic factors.

**Conclusion:**

About one-third of THA/TKA patients have low eHL, and low eHL was associated with poor QoL.

The aging population [1] is projected to increase the burden of osteoarthritis (OA), and the incidence of total hip and knee arthroplasty (THA/TKA) procedures worldwide is growing [2]. To avoid overburdening the healthcare system, patients are increasingly expected to manage their condition using digital health resources, including internet-delivered educational material and videoconferencing sessions with physiotherapists [3], cognitive behavioral programs [4], or smartphone applications for home exercise programs [5]. Communication with healthcare providers increasingly occurs digitally. To develop digital services that provide equal healthcare for all patients, it is essential to have knowledge regarding the competency within the specific patient group.

Digital health literacy refers to “the ability to seek, find, understand, and appraise health information from electronic sources and apply the knowledge gained to addressing or solving a health problem” [6]. Little is known about digital health literacy among patients with OA and THA/TKA. Such knowledge is important when tailoring health interventions and services to this patient group and it forms a basis for later studies.

To address this knowledge gap, we primarily aimed to describe digital health literacy levels in multiple domains by age and education among patients who have undergone hip or knee arthroplasty and secondarily to analyze how digital health literacy was related to their health related QoL, controlling for selected sociodemographic factors.

## Methods

This cross-sectional design study was planned and reported according to the STROBE guidelines [7].

### Participants

A sample of 800 patients, at least 18 years of age, who had undergone primary THA (n = 400) or TKA (n = 400) between 6 to 11 months prior, were randomly selected from the Norwegian Arthroplasty Register (NAR) in April 2022. A sample size of 800 was chosen based on an anticipated response rate of approximately 50%, and we intended to divide the sample into subgroups based on age, sex, and education level. This sample consisted of patients from all counties in Norway to match the Norwegian hip and knee arthroplasty population.

All selected patients received written information concerning the study, a written-consent form, and a paper questionnaire by mail between May and August 2022. Due to slow mail delivery and time constraints in the study, no reminder was sent. Those who wished to participate signed the consent form, filled in the questionnaire, and returned both in a sealed, opaque prepaid envelope.

### Measures

*Sociodemographic variables.* The sociodemographic data included age, sex, educational level, and type of surgery (hip/knee). For describing norm data, age was divided into 3 groups: younger age (< 65 years), medium age (65–74 years), or older age (≥ 75 years). In the other analysis, age was used as a continuous variable. Educational level was dichotomized as low = ≤ high school (level 0–4 according to International Standard Classification of Education 2011 [ISCED-11]) [8] or high = university (ISCED-11 level 5–8).

*Digital health literacy.* The eHealth Literacy Questionnaire (eHLQ) [9] was chosen in this study because it was developed based on the digital health literacy framework described by Norgaard et al. in 2015 [10], as it better reflects eHealth of today. We used the Norwegian version of the original eHLQ [9], which consists of 35 items assessing the 7 domains of the eHealth Literacy Framework: (i) using technology to process health information (Using technology, 5 items), (ii) understanding of health concepts and language (Understanding, 5 items), (iii) ability to actively engage with digital services (Engage, 5 items), (iv) feel safe and in control (Control, 5 items), (v) motivated to engage with digital services (Motivation, 5 items), (vi) access to digital services that work (Access, 6 items), and (vii) digital services that suit individual needs (Needs, 4 items). The original Danish version of eHLQ has satisfactory construct validity and reliability across a broad range of concepts in various groups [9]. Confirmatory factor analysis in a preliminary validity testing of the Norwegian version found that almost all factor loadings were high to acceptable [11]. All items are scored on a 4-point Likert scale ranging from 1 = strongly disagree to 4 = strongly agree, with higher scores indicating higher digital health literacy. Each domain is scored separately by summing the score on each item and dividing it by the number of items scored. If > 50% of the items in a domain were missing, a mean score was not calculated for that domain according to the guidelines for the original questionnaire. There is no consensus on what is “low” or” high” digital health literacy. Zangger et al. (2024) [12] have in concordance with the eHLQ developer Lars Kayser and the Region Zealand health Survey report [13] divided the scores into “insufficient” ≤ 2.5 and “sufficient” > 2.5. Based on this, we dichotomized the eHLQ score as low eHL ≤ 2.5 and high eHL > 2.5.

*Health-related Quality of Life and self-rated health.* Health-related QoL was measured using the EuroQol EQ-5D-5L [14], consisting of the EQ index and the EQ VAS. The EQ index includes 5 items assessing different dimensions of health status (mobility, self-care, usual activities, pain/discomfort, anxiety/depression). Each dimension is scored on a 5-point Likert scale with 5 categories from 1 = no problems to 5 = extreme problems and transformed into an index on a scale ranging from less than 0 (worse than dead) to 1 (no problems). The EQ VAS is a measure of self-rated health using a vertical visual analogue scale from 0 (“The worst health you can imagine”) to 100 (“The best health you can imagine”) [15]. The EQ-5D-5L is reliable and valid for this patient group [16].

### Statistics

Data were analyzed using the Statistical Package for the Social Sciences (SPSS; IBM Corp, Armonk, NY, USA) version 28 [17]. Descriptive statistics were used to describe the sample’s digital health literacy levels, sociodemographic characteristics, and health-related QoL. Digital health literacy norms by age group, sex, and educational level are presented as means, standard deviations (SD), and ranges. The proportion of patients with “low” digital health literacy is presented as number and percent, by age, level of education, and type of surgery. An independent-sample proportion test was used to explore the difference in proportions with low digital health literacy between age groups, levels of education, and type of operation. Correlations between the 7 digital health literacy domains and age, sex, and educational level were investigated using a Pearson product-moment correlation coefficient which can take a level between –1 and 1 where 0 refers to no correlation, –1 refers to perfect negative correlation (as one variable increases, the other decreases) and 1 refers to a perfect positive correlation (as one variable increases, so too does the other). Preliminary analyses were performed to ensure no violation of the assumptions of normality and linearity. Due to multiple testing the significance level was set to P = 0.01.

Univariable and separate multivariable linear regression models adjusting for selected sociodemographic factors (age, sex, education level, and type of surgery) were used to investigate how each of the digital health literacy domains was related to health-related QoL (EQ-5D-5L) and self-reported health (EQ VAS). Preliminary analyses were conducted to ensure no violation of the assumptions of normality, linearity, multicollinearity, and homoscedasticity. The 7 digital health literacy domains were strongly correlated to each other (Table 4), with most of the correlations exceeding 0.7. These correlations may suggest multicollinearity, which violates the assumptions for multivariable linear regression models. Therefore, for the multivariable regression models we decided to perform separate regression models for each dimension, while controlling for the relevant confounders.

### Ethics, registration, data sharing, funding, use of AI, and disclosures

The study has been performed in accordance with the ethical standards in the 1964 Declaration of Helsinki and the regulations of the US Health Insurance Portability and Accountability Act (HIPAA). The Regional Medical Research Ethics committee of Health East of Norway approved the study (2017/968). Written informed consent was obtained from all subjects.

AI was not used. All authors declare no conflict of interests. Complete disclosure of interest forms according to ICMJE are available on the article page, doi: 10.2340/17453674.2024.42304

## Results

### Response rate

404 patients consented to participate and returned the questionnaire. 21 of the responders had more than 50% missing values on the eHLQ and were excluded. The remaining 383 patients (48%) of the original sample were included in the analysis; 198 (52%) had TKA and 185 (48%) had THA (Figure).

**Figure:**
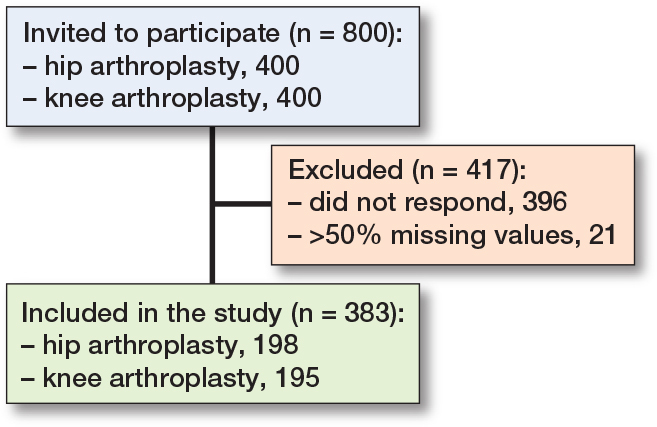
Flowchart of patient recruitment.

### Patient characteristics

Age, sex, and type of surgery of non-responders did not differ significantly from the responders (Table 1). Age ≥ 75 years and low education show the lowest digital health literacy score from 2.4 (SD 0.70) in “use technology,” to 3.1 (SD 0.38) in “control,” while age < 65 years and high education show highest digital health literacy score from 2.8 (SD 0.53) in “access,” to 3.2 (SD 0.59) in “engage.” “Control” has the highest score (3.2, SD 0.50) and “needs” has the lowest score (2.6, SD 0.65) regardless of age and education level (Table 2). The same tendency was found for both sexes (Tables 3 and 4, see Appendix).

**Table 1:** Sociodemographics and health-related quality of life score of responders (n = 383) and age, sex, and surgery of non-responders (n = 417)

Factor	Responders	Non-responders	P value
Age, mean (SD)	70 (9)	69 (11)	0.3
range	39–94	40–92	0.3
Age groups, n (%)			
< 65	98 (26)	127 (30)	
65–74	152 (40)	137 (33)	0.1
≥ 75	133 (34)	153 (37)	
Sex, n (%)			
Male	137 (36)	136 (33)	0.4
Female	246 (64)	281 (67)	
Operation, n (%)			
THA	185 (48)	214 (52)	0.4
TKA	198 (52)	203 (48)	
Education, n (%)			
Low	229 (60)		
High	151 (39)		
EQ Index, mean (SD)	0.88 (0.14)		
range	0.07–1.00		

^a^ Pearson’s chi-square.

THA = Total hip arthroplasty.

TKA = total knee arthroplasty.

Low education = ≤ high school (level 0–4 according to International

Standard Classification of Education 2011 (ISCED-11).

High education = > high school, university (ISCED-11 level 5–8).

**Table 2:** Digital Health Literacy Questionnaire mean scores by age group and level of education. Vakues are mean (SD) min–max

Domain	Education level ^a^		
Age	Low	High	All
1. Using technology to process health information			
< 65	2.8 (0.6) 1.4–4.0	3.1 (0.5) 2.2–4.0	3.0 (0.6) 1.4–4.0
65–74	2.7 (0.7) 1.0–4.0	2.9 (0.4) 1.6–3.8	2.8 (0.6) 1.0–4.0
≥ 75	2.4 (0.7) 1.0–4.0	2.6 (0.7) 1.0–3.8	2.5 (0.7) 1.0–4.0
All	2.7 (0.7) 1.0–4.0	2.9 (0.6) 1.0–4.0	2.7 (0.7) 1.0–4.0
2. Understanding of health concepts and language			
<65	3.0 (0.5) 1.8–4.0	3.2 (0.4) 2.4–4.0	3.1 (0.5) 1.8–4.0
65–74	3.0 (0.5) 1.0–4.0	3.1 (0.5) 2.2–4.0	3.0 (0.5) 1.0–4.0
≥75	2.8 (0.5) 1.8–4.0	3.0 (0.6) 1.0–4.0	2.9 (0.5) 1.0–4.0
All	2.9 (0.5) 1.0–4.0	3.1 (0.5) 1.0–4.0	3.0 (0.5) 1.0–4.0
3. Ability to actively engage with digital services			
<65	2.9 (0.7) 1.0–4.0	3.2 (0.6) 2.0–4.0	3.0 (0.7) 1.0–4.0
65–74	2.8 (0.7) 1.0–4.0	3.2 (0.5) 1.8–4.0	3.0 (0.6) 1.0–4.0
≥75	2.5 (0.7) 1.0–4.0	2.7 (0.8) 1.0–4.0	2.6 (0.7) 1.0–4.0
All	2.7 (0.7) 1.0–4.0	3.1 (0.6) 1.0–4.0	2.9 (0.7) 1.0–4.0
4. Feel safe and in control			
<65	3.2 (0.5) 2.2–4.0	3.1 (0.5) 1.4–4.0	3.2 (0.5) 1.4–4.0
65–74	3.2 (0.5) 1.8–4.0	3.2 (0.6) 1.0–4.0	3.2 (0.5) 1.0–4.0
≥75	3.1 (0.4) 2.0–4.0	3.0 (0.6) 1.0–4.0	3.1 (0.5) 1.0–4.0
All	3.2 (0.5) 1.8–4.0	3.1 (0.6) 1.0–4.0	3.1 (0.5) 1.0–4.0
5. Motivated to engage with digital services			
<65	2.9 (0.6) 1.75–4.0	2.9 (0.4) 2.2–4.0	2.9 (0.5) 1.8–4.0
65–74	2.8 (0.6) 1.0–4.0	2.9 (0.5) 2.0–4.0	2.8 (0.6) 1.0–4.0
≥75	2.5 (0.7) 1.0–4.0	2.6 (0.6) 1.0–4.0	2.5 (0.6) 1.0–4.0
All	2.7 (0.7) 1.0–4.0	2.8 (0.5) 1.0–4.0	2.7 (0.6) 1.0–4.0
6. Access to digital services that work			
<65	2.9 (0.6) 1.8–4.0	2.7 (0.5) 1.5–4.0	2.9 (0.6) 1.5–4.0
65–74	2.9 (0.6) 1.0–4.0	2.9 (0.4) 2.0–3.8	2.9 (0.5) 1.0–4.0
≥75	2.6 (0.6) 1.2–4.0	2.6 (0.6) 1.0–3.8	2.6 (0.6) 1.0–4.0
All	2.8 (0.6) 1.0–4.0	2.8 (0.5) 1.0–4.0	2.8 (0.6) 1.0–4.0
7. Digital services that suit individual needs			
<65	2.8 (0.6) 1.3–4.0	2.8 (0.5) 1.8–4.0	2.8 (0.6) 1.3–4.0
65–74	2.7 (0.7) 1.0–4.0	2.9 (0.6) 1.5–4.0	2.8 (0.6) 1.0–4.0
≥75	2.4 (0.7) 1.0–4.0	2.4 (0.6) 1.0–4.0	2.4 (0.6) 1.0–4.0
All	2.6 (0.7) 1.0–4.0	2.7 (0.6) 1.0–4.0	2.6 (0.6) 1.0–4.0

a Education level: See Table 1.

**Table 3:** Male eHLQ^a^ mean scores by age group and level of education. Values are mean (SC) min–max

Domain	Age	Education level	
		low	high
1. Using technology	<65	3.0 (0.7) 2.0–4.0	3.0 (0.5) 2.4–4.0
	65–74	2.5 (0.6) 1.0–3.6	3.0 (0.4) 2.2–3.8
	≥75	2.5 (0.7) 1.0–4.0	2.6 (0.9) 1.0–3.8
2. Understand	<65	3.2 (0.5) 2.4–4.0	3.2 (0.4) 2.6–4.0
	65–74	2.8 (0.5) 1.0–3.8	3.2 (0.4) 2.4–4.0
	≥75	2.9 (0.5) 2.0–4.0	3.0 (0.7) 1.0–4.0
3. Engage	<65	3.0 (0.8) 1.0–4.0	3.3 (0.6) 2.2–4.0
	65–74	2.6 (0.5) 1.6–3.6	3.2 (0.4) 2.0–4.0
	≥75	2.5 (0.7) 1.0–4.0	2.9 (0.8) 1.0–3.8
4. Control	<65	3.3 (0.5) 2.4–4.0	3.1 (0.5) 2.2–4.0
	65–74	3.1 (0.5) 1.8–4.0	3.2 (0.4) 2.4–4.0
	≥75	3.1 (0.3) 2.4–4.0	3.1 (0.7) 1.0–4.0
5. Motivation	<65	3.1 (0.7) 1.8–4.0	2.9 (0.5) 2.2–4.0
	65–74	2.6 (0.6) 1.0–3.4	3.0 (0.5) 2.2–4.0
	≥75	2.6 (0.7) 1.0–4.0	2.6 (0.8) 1.0–4.0
6. Access	<65	3.1 (0.6) 1.8–4.0	2.8 (0.5) 1.8–4.0
	65–74	2.7 (0.6) 1.0–3.8	3.0 (0.2) 2.0–3.8
	≥75	2.6 (0.6) 1.4–4.0	2.6 (0.8) 1.0–3.7
7. Needs	<65	3.0 (0.7) 1.3–4.0	2.9 (0.4) 2.3–4.0
	65–74	2.5 (0.6) 1.0–3.5	2.9 (0.5) 2.0–4.0
	≥75	2.4 (0.8) 1.0–4.0	2.6 (0.8) 1.0–4.0

a eHLQ = digital Health Literacy Questionnaire

**Table 4:** Female eHLQ a mean scores by age group, level of education

Domain	Age	Education level	
		low	high
1. Using technology	<65	2.7 (0.6) 1.4–4.0	3.1 (0.5) 2.2–4.0
	65–74	2.9 (0.7) 1.0–4.0	2.9 (0.5) 1.6–3.8
	≥75	2.4 (0.7) 1.0–4.0	2.6 (0.7) 1.0–3.8
2. Understand	<65	2.9 (0.5) 1.8–3.6	3.2 (0.4) 2.4–3.8
	65–74	3.1 (0.5) 2.2–4.0	3.1 (0.5) 2.2–4.0
	≥75	2.9 (0.5) 1.8–4.0	3.0 (0.5) 1.8–4.0
3. Engage	<65	2.8 (0.7) 1.2–4.0	3.2 (0.6) 2.0–4.0
	65–74	2.9 (0.7) 1.0–4.0	3.2 (0.5) 1.8–4.0
	≥75	2.5 (0.7) 1.0–3.6	2.7 (0.7) 1.0–4.0
4. Control	<65	3.1 (0.5) 2.2–4.0	3.1 (0.6) 1.4–4.0
	65–74	3.3 (0.5) 1.8–4.0	3.1 (0.7) 1.0–4.0
	≥75	3.1 (0.4) 2.0–4.0	3.0 (0.5) 1.8–4.0
5. Motivation	<65	2.7 (0.5) 1.8–4.0	2.9 (0.4) 2.2–3.8
	65–74	2.9 (0.6) 1.2–4.0	2.9 (0.5) 2.0–3.8
	≥75	2.5 (0.7) 1.0–4.0	2.6 (0.5) 1.2–3.8
6. Access	<65	2.8 (0.5) 1.8–4.0	2.7 (0.5) 1.5–3.5
	65–74	3.0 (0.6) 1.7–4.0	2.9 (0.4) 2.0–3.7
	≥75	2.6 (0.6) 1.2–4.0	2.6 (0.6) 1.5–3.8
7. Needs	<65	2.6 (0.5) 1.5–3.8	2.7 (0.5) 1.8–3.5
	65–74	2.8 (0.7) 1.3–4.0	2.9 (0.6) 1.5–4.0
	≥75	2.4 (0.6) 1.0–4.0	2.3 (0.5) 1.0–3.3

^a^ eHLQ = digital Health Literacy Questionnaire

46% of the responders did not agree that the digital services suit individual needs (domain 7) while only 7.7% did not agree that they feel safe and in control (domain 4) (Table 5). There was no difference between THA and TKA patients except for domain 1 (Using technology) where more patients with TKA had a low score.

**Table 5:** Differences in proportions with low digital health literacy (≤ 2.5) by age, education level, and type of surgery

Domain	n (%)	n (%)	95% CI of the difference	Total n (%)
1. Using technology to process health information				130 (34)
Age < 65 vs ≥ 65	26 (6.9)	104 (27)	–0.10 to 0.06	
Low vs high edu.	93 (41)	35 (23)	0.08 to 0.27	
THA vs TKA	52 (29)	78 (40)	–0.20 to –0.15	
2. Understanding of health concepts and language				54 (14)
Age < 65 vs ≥ 65	19 (5.0)	42 (11)	–0.20 to –0.01	
Low vs high edu.	39 (17)	15 (10)	0.001 to 0.14	
THA vs TKA	23 (13)	31 (16)	–0.10 to 0.04	
3. Ability to actively engage with digital services				105 (28)
Age < 65 vs ≥ 65	19 (5.0)	86 (23)	–0.20 to –0.01	
Low vs high edu.	79 (35)	26 (17)	0.09 to 0.26	
THA vs TKA	48 (26)	57 (29)	–0.11 to 0.07	
4. Feeling safe and in control				29 (7.7)
Age < 65 vs ≥ 65	9 (2.4)	20 (5.3)	–0.04 to 0.09	
Low vs high edu.	3 (5.8)	16 (11)	–0.11 to 0.01	
THA vs TKA	13 (7.1)	16 (8.2)	–0.07 to 0.04	
5. Motivated to engage with digital services				130 (35)
Age < 65 vs ≥ 65	26 (6.9)	104 (28)	–0.21 to –0.003	
Low education	85 (38)	43 (38)	–0.003 to 0.19	
THA	62 (34)	68 (35)	–0.10 to 0.09	
6. Access to digital services that work				124 (33)
Age < 65 vs ≥ 65	29 (7.7)	95 (25)	–0.15 to 0.07	
Low vs high edu.	71 (32)	52 (34)	–0.13 to 0.07	
THA vs TKA	62 (34)	62 (32)	–0.07 to 0.12	
7. Digital services that suit individual needs				171 (46)
Age < 65 vs ≥ 65	36 (9.6)	135 (36)	–0.23 to –0.01	
Low vs high edu.	11 (50)	59 (39)	0.01 to 0.21	
THA vs TKA	83 (46)	88 (45)	–0.09 to 0.11	

For abbreviations, see Table 1 and CI = confidence interval.

### Correlations

The correlations between the digital health literacy domains and age, sex, educational level, and health-related QoL showed no significant correlations between sex and the digital health literacy domains (Table 6). Age was negatively correlated (P < 0.01) with all digital literacy domains except domain 4 (Control). Educational level was positively correlated with digital health literacy domains 1 (Using technology), 2 (Understanding), and 3 (Engage). EQ index was positively correlated with domain 3 (Engage) and 4 (Control), 6 (Access), and 7 (Needs). The correlation was small according to the guidelines suggested by Cohen (1988) [18] (small = 0.10–0.29, medium = 0.30–0.49, large = 0.50–1.00).

**Table 6:** Pearson correlations between eHealth Literacy Questionnaire (eHLQ) score and age, sex, education, and EQ VAS

	Age	Female	Education	eHLQ Domain^a^						
				1	2	3	4	5	6	7
Female	0.04									
Education	–0.18	–0.04								
eHLQ Domain ^a^										
1	–0.29	–0.01	0.21							
2	–0.17	–0.00	0.19	0.75						
3	–0.30	–0.04	0.29	0.87	0.70					
4	–0.09	–0.01	–0.05	0.47	0.59	0.45				
5	–0.21	–0.05	0.09	0.85	0.75	0.74	0.56			
6	–0.17	–0.04	–0.01	0.76	0.67	0.70	0.65	0.81		
7	–0.24	–0.06	0.09	0.81	0.67	0.78	0.55	0.85	0.84	
EQ VAS ^b^	0.10	–0.07	0.13	0.13	0.12	0.23	0.08	0.11	0.13	0.16
EQ index	0.03	–0.02	0.12	0.07	0.10	0.17	0.14	0.07	0.11	0.12

a For eHLQ domains, see Table 2.

b EQ VAS = self-reported health on a 0–100 visual analogue scale.

Correlation > 0.13 or < –0.13 is significant (P < 0.01; 2-tailed).

### Multivariable linear regression analysis

Results from the separate multivariable linear regression analysis showed that digital health literacy domain 1 (Using technology), 3 (Engage), 4 (Control), 6 (Access), and 7 (Needs) were positively associated with health-related QoL, when adjusted for patients’ age, sex, education level, and type of surgery (Table 7). The strongest association was found in domain 3 (Engage) and 4 (Control), where the unstandardized coefficient (B) shows that for each unit change in eHLQ there will be 0.04 unit change in EQ Index. The association between digital health literacy and EQ VAS is shown in Table 8 (see Appendix) and demonstrated associations with most domains, with the strongest association with domain 3 (Engage).

**Table 7:** Linear regression analyses of associations between the eHealth literacy domains and health-related quality of life with EQ index as dependent variable

eHLQ domain	Univariable regression coefficients adjusted for covariates		Separate multivariable regression coefficients	
	B (CI)	Beta	B (CI)	Beta
1. Using technology	0.02 (–0.01 to 0.04)	0.07	0.01 (0.01 to 0.04)	0.07
2. Understand	0.03 (–0.001 to 0.06)	0.10	0.02 (–0.01 to 0.05)	0.08
3. Engage	0.03 (0.01 to 0.06)	0.17	0.04 (0.01 to 0.06)	0.18
4. Control	0.04 (0.01 to 0.07)	0.14	0.04 (0.02 to 0.07)	0.15
5. Motivation	0.02 (–0.01 to 0.04)	0.07	0.02 (–0.01 to 0.04)	0.07
6. Access	0.03 (0.001 to 0.05)	0.11	0.03 (0.004 to 0.06)	0.12
7. Needs	0.03 (0.003 to 0.05)	0.12	0.03 (0.01 to 0.05)	0.13
Covariates				
Age	0.0005 (–0.001 to 0.002)	0.03		
Sex (male = 0, female = 1)	–0.01 (–0.04 to 0.03)	–0.02		
Education	0.03 (–0.004 to 0.06)	0.09		
Surgery (THA = 1, TKA = 2)	–0.03 (–0.06 to –0.001)	–0.10		

B = The unstandardized coefficient B: change in EQ Index by each unit change in eHLQ score with 95% confidence intervals (CI).

Beta = standardized beta coefficients.

Abbreviations, see Table 1 and eHLQ = eHealth Literacy Questionnaire.

For eHLQ domains, see Table 2.

**Table 8:** Linear regression analyses of associations between the eHealth literacy domains and self-reported health with EQVAS as dependent variable

eHLQ domain	Univariable regression coefficient adjusted for covariates		Separate multivariable regression coefficients	
	B (CI)	Beta	B (CI)	Beta
1. Using technology	3.2 (0.79 to 5.7)	0.13	3.8 (1.2 to 6.4)	0.16
2. Understand	3.9 (0.65 to 7.2)	0.12	3.8 (0.47 to 7.1)	0.12
3. Engage	5.3 (3.0 to 7.6)	0.23	6.1 (3.7 to 8.5)	0.26
4. Control	2.4 (–0.87 to 5.7)	0.08	3.0 (–0.23 to 6.3)	0.09
5. Motivation	3.0 (0.30 to 5.8)	0.11	3.5 (0.75 to 6.3)	0.13
6. Access	3.6 (0.76 to 6.5)	0.13	4.3 (1.5 to 7.1)	0.15
7. Needs	4.1 (1.6 to 6.6)	0.16	4.8 (2.2 to 7.3)	0.19
Covariates				
Age (< 65 = 1, ≥ 65 = 2)	0.18 (–0.004 to 0.36)	0.10		
Sex (male = 0, female = 1)	–2.3 (–5.7 to 1.2)	–0.07		
Education (low = 1, high = 2)	3.8 (0.42 to 7.1)	0.11		
Surgery (THA = 1, TKA = 2)	–3.2 (–6.4 to 0.11)	–0.10		

For Abbreviations, see Table 5.

## Discussion

We aimed to describe digital health literacy among patients who had undergone hip or knee arthroplasty and to analyze how digital health literacy was related to their health related QoL. It was found that digital health literacy in this population varied by age and educational level, with younger patients with high educational level having the highest digital health literacy score. Health-related QoL was associated with some of the digital health literacy domains.

To the authors’ knowledge, norm data for digital health literacy was not available for THA/TKA patients prior to our study. A scoping review by Wang and Luan (2022) [19] summarized that digital health literacy among older adults was lower in those with lower education levels. This is in accordance with the findings in our study. Cherid et al. (2020) [20] showed in their study on people ≥ 50 years with recent fractures that there was no difference in digital health literacy between the male and female patients or between age groups of 50–64 years and 65–74 years, while the age group over 75 years had lower digital health literacy. However, they did not account for education level. We have described digital health literacy in 3 age categories (< 65, 65–74, and ≥ 75) by education level and sex. Our data can therefore be used to compare digital health literacy with other studies across various age groups, sex, and educational levels. The results from our study can form the basis for observing changes in digital health literacy over time.

Our study demonstrated an association between health-related QoL and some domains of digital health literacy. This is similar to what Filabadi et al. (2020) [21] showed in their study on 400 clients of different community health centers in Teheran, aged 17–75 years, where they found that digital health literacy was positively correlated with patients’ health-related QoL. The relationship between digital health literacy and health-related QoL demonstrated in our study is valuable knowledge when developing interventions tailored to improve health-related QoL in the population. By enhancing digital health literacy and tailoring services and treatment to the health literacy of the specific patient group, this can contribute to improving the quality of life within that group.

Norway is a country with a high degree of digitalization in the society. 9 out of 10 use the BankID, which is an electronic signature solution, and in 2020 80% of Norwegian citizens were active users of the national health portal (Helsenorge.no) to gain access to healthcare services, communicate with health professionals, and get access to health information. Holt et al. (2019) [22] showed that active users of corresponding services in Denmark had higher digital health literacy than non-users. The high degree of digitalization in society may result in higher digital skills among the citizens. The European survey described great differences between European countries [23]. Thus, the results in our study may not be generalizable to other countries with a lower grade of digitalization.

### Strengths

We included a large number of participants randomly selected from the Norwegian Arthroplasty Register (NAR) representing all counties in Norway. Sociodemographic variables matched all THA and TKA patients registered in the NAR [24], and the distribution of age and sex in non-responders was not different from that of the responders.

Another advantage was that the questionnaires were on paper and sent by regular mail, thus not excluding individuals who do not have access to a digital device or those with low digital competence.

### Limitations

Although all counties in Norway were represented and the age and sex distribution in our study was similar to all patients registered in NAR, it is possible that this sample was not representative of the entire Norwegian THA/TKA population with regard to other variables such as education or physical status.

The response rate in this study was 48%. According to a recent review by Edwards et al. (2023) [25] contacting patients in advance, sending reminder letters, or offering an incentive to patients who respond can increase response rate. However, due to limited time and resources, we were unable to apply these methods. To achieve the highest possible response rate, we emphasized making the questionnaire as short as possible, providing an explanation in simple language, and including a prepaid return envelope. The low response rate may have influenced the representativeness. Cognitive function declines with increasing age in the general population and among patients with osteoarthritis [26]. We have not tested cognitive function in this population. We also do not have information on the education level of the non-responder group. It is possible that patients with reduced cognitive function and low educational level are overrepresented in the non-responder group, hence affecting representativeness.

We examined digital health literacy in patients who had undergone total joint arthroplasty 6–11 months previously. In another study, general health literacy in TKA patients increased from before surgery to 3 and 6 months after surgery [27]. Hence, the results from our study may not be representative for OA patients without knee and hip arthroplasty.

The disadvantage of the eHLQ is that it has not been as thoroughly tested for psychometric properties as the eHEALS instrument. However, recent tests conducted in Denmark, Sweden, and Norway show the properties to be good [9,11,28].

Another limitation is that we used only self-reported data to measure the patients’ digital health literacy. Self-reported competence may not reflect the patients’ actual competence. Additionally, patients with low digital health literacy might be over- or under-represented among non-responders, even though we used paper questionnaires.

### Conclusion

About one-third of THA/TKA patients have low eHL, and low eHL was associated with poor QoL.

*In perspective,* the goal is to offer equal health treatment and service to all patients. The findings from our study are useful for clinical practice and the development of future interventions and services. In the clinic, it may be beneficial to assess patients’ digital health literacy to tailor services according to their competencies and to offer support for the use of digital services to those with low digital health literacy, ensuring that there are non-digital alternatives. Nearly half of the patient group in this study reported that digital services do not suit their needs. This should have implications for how we develop new digital services, for example, by actively involving user representatives throughout the entire development process. Future studies may investigate whether improving digital health literacy levels might contribute to improved health-related QoL in this patient group.

## Notes

TR: conceptualization, methodology, writing the original draft. IKN, IBB: data collection, writing—review & editing. AMF: data collection, writing—statistics, review & editing. OF, STS: supervision, writing—review & editing. AL: conceptualization, methodology, writing—review & editing. MFL: conceptualization, methodology, statistics, supervision, writing—review & editing. MB: supervision, writing the original draft.

This paper is a product stemming from the Norwegian research project “The MultiKnee trial.” Dr Maren Falch Lindberg is the principal investigator (PI) and Drs Anners Lerdal and Arild Aamodt are Co-PIs. Ingvild Buset Bergvad, Alexander Eikrem-Lüthi, and Turid Rognsvåg are PhD students supervised by their seniors Drs Anners Lerdal, Jon Magnussen, Maren Falch Lindberg, Mona Badawy, Ove Furnes, and Søren T. Skou. The other members of the Multi-knee research team are Drs Caryl L. Gay, Stig Heir, Inger Holm, Nina Kise, Tor Kjetil Nerhus, Milada C. Småstuen, and Jan Stubberud. Katrine Rutledal, deputy director for Lovisenberg User Board, provided user participation.

Handling co-editors: Li Felländer-Tsai and Robin Christensen

*Acta* thanks Leif Rune Hedman and other anonymous reviewers for help with peer review of this manuscript.
